# Morphants: A New Systematic Vertebrate Functional Genomics Approach

**DOI:** 10.1002/1097-0061(200012)17:4<302::AID-YEA53>3.0.CO;2-#

**Published:** 2006-04-25

**Authors:** Stephen C. Ekker

**Affiliations:** ^1^ Arnold and Mabel Beckman Center for Transposon Research at the University of Minnesota, Department of Genetics, Cell Biology and Development, 6-160 Jackson Hall, 321 Church St SE, Minneapolis, MN 55455, USA

## Abstract

The vertebrate genome contains a predicted 50 000 100 000 genes, many of unknown function. The recent development of morpholino-based gene knock-down technology in zebrafish has opened the door to the genome-wide assignment of function based on sequence in a model vertebrate. This review describes technical aspects of morpholino use for functional genomics applications, including the potential for multigene targeting and known methodological limitations. The result of successful gene inactivation by this agent is proposed to yield embryos with a ‘morphant’ phenotypic designation. The establishment of a morphant database opens the door to true functional genomics using the vertebrate, *Danio rerio*.


**Yeast**
*Yeast* 2000; **17**: 302–306.

**Review Article**

### Morphants: a new systematic vertebrate functional genomics approach

Stephen C. Ekker*
*Arnold and Mabel Beckman Center for Transposon Research at the University of Minnesota, Department of Genetics, Cell Biology and Development, 6-160 Jackson Hall, 321 Church St SE, Minneapolis, MN 55455, USA*

*Correspondence to: Stephen C. Ekker, Arnold and Mabel Beckman Center for Transposon Research at the University of Minnesota, Department of Genetics, Cell Biology and Development, 6-160 Jackson Hall, 321 Church St SE, Minneapolis, MN 55455, USA. E-mail: ekker001@mail.med.umn.edu*

Received: 9 October 2000
Accepted: 12 October 2000

### Abstract

**The vertebrate genome contains a predicted 50 000–100 000 genes, many of unknown function. The recent development of morpholino-based gene knock-down technology in zebrafish has opened the door to the genome-wide assignment of function based on sequence in a model vertebrate. This review describes technical aspects of morpholino use for functional genomics applications, including the potential for multigene targeting and known methodological limitations. The result of successful gene inactivation by this agent is proposed to yield embryos with a 'morphant' phenotypic designation. The establishment of a morphant database opens the door to true functional genomics using the vertebrate, *Danio rerio*. Copyright © 2000 John Wiley & Sons, Ltd.**

**Keywords:** zebrafish; morpholino; morphant; vertebrate; functional genomics

### Introduction

The ability of the various genome projects to acquire gene sequence data has far outpaced our ability to ascribe biological functions to these new genes. How can we determine which of the increasing number of these uncharacterized gene products are involved in a given biological or disease process? This dilemma has led to the concept of a scientific field called 'functional genomics', which can be defined as the attempt to match biological function with gene sequence on a genome scale. For the many biological processes that are well conserved in evolution, model systems with rapid genetic tools, such as *Drosophila melanogaster*, have opened the door to functional genomics. In this paradigm, genes with specific biological roles are identified first in the model organism and genome databases are subsequently used to identify human homologues. The *hedgehog* and *wnt* signalling cascades are excellent examples of conserved pathways first worked out in invertebrate model systems, with significant implications on health and disease upon analysis of the human pathway equivalents.

Many biological and biochemical pathways are not conserved between worms, flies and vertebrates and, consequently, studies directly using biologically more complex model systems, such as the mouse and the fish, are warranted. Examples include neural crest formation, most organogenesis pathways, and signalling cascades such as that induced by vascular endothelial growth factor (VEGF). Indeed, studies with these more closely related model systems are required to understand many critical details of even conserved pathways and their implications on human health and disease. For example, the relationship between holoprosencephaly and *hedgehog* signalling was not clarified until work was performed in the fish, the mouse and in humans, despite the fundamental role of this pathway in the development of most animals on this planet [23].

### The zebrafish

The results of two recent, very large and very expensive 'saturation screens' in zebrafish using chemical mutagenesis demonstrate the advantage of this model organism for the identification of

Copyright © 2000 John Wiley & Sons, Ltd.

genes required for vertebrate-specific processes [4,6]. Of the hundreds of developmental loci identified, however, less than 50 have been molecularly characterized because of the difficult and time-consuming nature of positional cloning and related methods [21]. Forward genetics in zebrafish identifies novel genes of interest but is limited by the laborious nature of the F3 genetic screens and the work required for molecular identification of the mutant locus. Similar limitations apply to current chemical mutagenesis projects now under way in a variety of academic and commercial mouse laboratories.

Insertional mutagenesis methods using retroviruses are also currently used in zebrafish genetics research [21]. This approach reduces the work required for the molecular assignment of function to sequence, but this tool is also significantly lower in mutagenesis effectiveness compared to chemical agents. In addition, these screens currently require analyses of F3 embryos, requiring a significant commitment to research infrastructure and animal facilities. The laboratory of Dr N. Hopkins is in the final stages of a large insertional screen, and the expected outcome is the assignment of sequence to function of ca. 250–500 genes [1]. Other insertional mutagens based on transposons are under development [9,17]. Transposons have the potential for performing F1 screens based on insertion site context (through gene or enhancer 'trapping'), but their efficacy in a genome-wide screen is still unknown.

An alternative to fish or mouse forward genetic screens employs mouse ES cell technology in a reverse genetic approach. In this strategy, the sequence of an individual gene is altered or removed from the chromosome of murine ES cells and the gene's function directly determined, using specific developmental assays in mice carrying deficiencies in both targeted chromosomes generated from these ES cells. This approach is limited by the significant cost ($\geq$\$100 000 per gene) and time ($\geq$1 year) required for the F3 mouse genetics work.

### Morpholinos

An ideal gene targeting technique should be specific, straightforward to perform, competent for action in all cell types, efficient at depleting the selected protein, amenable to the targeting of many genes, have little or no non-specific effects, and be reproducible. RNA-based interference (RNAi) [10,22; for review, see 8] can often satisfy many of these requirements in the nematode and *Drosophila*. RNAi has been reported to work in zebrafish, with highly variable and controversial results [11,13,24], however. Other methods, including RNAse-H-mediated strategies, have been tried with only modest success [3]. This author wonders whether the effectiveness of RNAi to specifically remove gene function in mice [20,25,26] will be subject to the same potential ambiguities in targeting noted for zebrafish [13] and *Xenopus* (Strege P, Ekker SC, unpublished observations).

Another strategy to inhibit gene function that has been recently shown to be extremely effective in zebrafish embryos [12] employs the gene-targeting agents called morpholinos as sequence-specific translational inhibitors. Morpholinos are chemically modified oligonucleotides with similar base stacking abilities as natural genetic material but have a morpholine moiety instead of a ribose [18,19]. In addition, a phosphorodiamidate linkage is used, resulting in a neutral charge backbone. These two modifications form a modified and highly soluble polymer capable of hybridizing single-stranded nucleic acid sequences with high affinity and little cellular toxicity and are free of most or all non-specific side effects [18,19]. Indeed, morpholinos are not subject to any known endogenous enzymatic degradation activity. Morpholinos have been shown to bind to and block translation of mRNA both *in vitro* and in tissue culture [18,19]. In contrast to traditional antisense oligonucleotide approaches that utilize RNAse-H-based degradation of mRNA as a mechanism of action, morpholinos appear to function through the hindrance of translational initiation [18,19]. This alternative antisense approach makes morpholino targeting highly predictable for oligo design and significantly reduces non-specific effects. Recent results *in vivo* have demonstrated the potential of this reagent for therapeutic and genomics applications [2,7,12,16]. The use of morpholinos in zebrafish have shown these compounds to be (a) sequence specific and (b) extremely potent in all cells during the first 50 h of development in F0 zebrafish embryos as targeted gene 'knock-down' agents [12]. This time period in the zebrafish embryo includes the fundamental vertebrate processes of segmentation and organogenesis. This tool

Copyright © 2000 John Wiley & Sons, Ltd. 

offers the opportunity to pursue sequence specific gene targeting studies without the necessity of laborious, time-consuming and expensive F3 vertebrate genetic testing. Morpholinos thus offer a high throughput F0 vertebrate assay system for vertebrate functional genomics applications.

### Known limitations of morpholino-based gene targeting

The major assumption to the use of morpholinos as gene-targeting agents is the success rate for specific gene inactivation. We generated morpholinos against known genes to determine an estimate of success rate. We have targeted *shh*, *chordin*, *no tail*, *one-eyed-pinhead (oep)*, *sparse*, *nacre*, *urod*, *bozozok/dharma*, an EF1a–GFP transgene, *pax 2.1*, *bmp1*, *bmp2b*, *bmp7*, *alk8*, *smad5*, *wnt5* and *wnt11* (12; Nasevicius A, Ekker SC, unpublished observations; Hammerschmidt M, personal communication). With only one recent exception (*pax 2.1*), all genes targeted resulted in clear specific gene inactivation with the first morpholino tried (16/17 or >94% success rate). This is an upper estimate of the expected rate against unknown genes. The effective rate will be reduced by morpholino mistargeting and any other non-specific effects. In some instances, a morpholino will also inhibit a second gene and result in embryos with a combined phenotype. An extreme example is represented by the *bozozok/dharma* morpholino, in which a second effect (CNS degeneration) is superimposed on the *bozozok* loss of function phenotype [12]. If these secondary, non-specific effects result in loss of embryonic structures or premature death of the embryo, then the function of the gene of choice will not be scorable using this technology. The reduction of morpholino success rate by this mistargeting rate (2/17) is thus a lower estimate of morpholino screening efficiency, yielding an initial morpholino screening rate of 82% (14/17). A significant fraction of these 'missed' genes can be recovered by the use of a second morpholino of unrelated sequence. Assuming a similar specific success rate of ca. 80% for the remaining 18% of 'missed' genes yields an additional 14% of genes potentially targeted by morpholinos, for a combined expected success rate of >95% for genes screened using two targeted morpholinos.

Potential explanations for some missed targets include a failure to inhibit the translation of genes with complex promoters – transcripts with multiple leader sequences – or to a failure to bind and inactivate a selected gene due to differences in genetic background. Should the leader sequence in a specific locus be especially prone to polymorphism, the selected gene might not be inactivated in all embryos due to the high specificity of morpholino targeting *in vivo*. The *one-eyed-pinhead* locus is a potential example of this phenomenon [12]; in one wild-type strain, only ca. 50% of embryos responded to this morpholino; in another, none. Other, less direct strain differences could also reduce the effectiveness of morpholinos. For example, variations in genetic backgrounds could alter the penetrance of a given morpholino effect due to genetic factors in a second, modulator locus. The characterization and inclusion of common 'wild-type' and other non-isogenic laboratory strains in the sequencing project is suggested to make maximum use of morpholino technology in zebrafish.

As with most genetic screens, morpholinos are also limited by functional redundancy, an issue especially relevant to vertebrates. Moreover, the zebrafish genome contains an additional set of incomplete duplicates for an estimated 30% of genes found in mammals [14,15]. This partial genome duplication occasionally results in two orthologues in zebrafish for one in humans. One example is the *sonic hedgehog* locus [12]. Comparative expression profiles of likely orthologues, however, demonstrate only a duplication of a subset of expression patterns for these genes. Indeed, no two orthologues have identical expression patterns in zebrafish [5]. The use of morpholinos, however, is highly amenable to rapid tests of redundancy through the simultaneous targeting of genes of related sequence (see *shh* and *twhh* double knockdowns [12]). Multigene targeting strategies are thus practical using current morphino technology, with an estimated minimum success rate of $(0.82 \times 0.82) = 67\%$. For genes amenable to this strategy, morpholinos will be extremely effective at identifying and testing molecules with redundant functions *in vivo*.

### Morphants

By analogy to the term 'mutant', which describes animals in which a specific gene has been altered

Copyright © 2000 John Wiley & Sons, Ltd. 

through mutation of the locus, we propose the term 'morphant' to describe animals in which a selected gene has been successfully inactivated by morpholino targeting. This simplification retains the distinction between classical genetic approaches to loss of function and morpholino-based targeting strategies. In addition, the standard for describing a 'morphant' effect should be only after the results are confirmed through either mRNA rescue or targeting using a second morpholino of unrelated sequence.

An example of the use of this term can be found in our compiled nascent morphant database (**http://beckmancenter.ahc.umn.edu/**). Just as there is now a zebrafish stock centre that carries many zebrafish mutations, we now carry the sequence and aliquots of morpholinos that yield specific morphant effects for request by the zebrafish community. A single, standard (300 nmol) morpholino synthesis generates sufficient reagent for use by 50 or more labs interested in testing for a possible role of a particular gene in their selected biological process. This will be of value for genes with known mutations as well; analyses using morphant phenocopies can be an initial screening tool that does not require the care and maintenance of fish stocks that may or may not be of interest to that investigator. We will accept both sequence and reagent aliquots from labs interested in supporting this effort, and we plan to update and distribute the status of this database regularly with unpublished morphants, using the Zebrafish Science Monitor. We will support this effort while there is expressed interest or until this database has been incorporated as a more formal part of the community infrastructure. In the future, the ability to screen a morphant database systematically for all genes involved in a particular biological process may move vertebrate functional genomics from the virtual to the real world.

### Acknowledgements

I wish to thank A. Nasevicius and S. Sumanas for critical reviews of this manuscript. Many thanks to numerous members of the zebrafish community for their support and independent confirmation of this methodology. I thank M. Hammerschmidt for the communication of results prior to publication. This work was supported in part by the National Institutes of Health, Grant No. GM55877, to SCE.

### References

1. Amsterdam A, Burgess S, Golling G, *et al*. 1999. A large-scale insertional mutagenesis screen in zebrafish. *Genes Dev* **13**: 2713–2724.
2. Arora V, Knapp DC, Smith BL, *et al*. 2000. c-Myc antisense limits rat liver regeneration and indicates role for c- Myc in regulating cytochrome P-450 3A activity. *J Pharmacol Exp Ther* **292**: 921–928.
3. Barabino SM, Spada F, Cotelli F, Boncinelli E. 1997. Inactivation of the zebrafish homologue of Chx10 by antisense oligonucleotides causes eye malformations similar to the ocular retardation phenotype. *Mech Dev* **63**: 133–143.
4. Driever W, Solnica-Krezel L, Schier AF, *et al*. 1996. A genetic screen for mutations affecting embryogenesis in zebrafish. *Development* **123**: 37–46.
5. Gates MA, Kim L, Egan ES, *et al*. 1999. A genetic linkage map for zebrafish: comparative analysis and localization of genes and expressed sequences. *Genome Res* **9**: 334–347.
6. Haffter P, Granato M, Brand M, *et al*. 1996. The identification of genes with unique and essential functions in the development of the zebrafish, *Danio rerio*. *Development* **123**: 1–36.
7. Heasman J, Kofron M, Wylie C. 2000. ß-catenin signalling activity dissected in the early Xenopus embryo: a novel antisense approach. *Dev Biol* **222**: 124–134.
8. Hunter CP. 2000. Shrinking the black box of RNAi. *Curr Biol* **10**: R137–R140.
9. Ivics Z, Hackett PB, Plasterk RH, Izsvak Z. 1997. Molecular reconstruction of Sleeping Beauty, a Tc1-like transposon from fish, and its transposition in human cells. *Cell* **91**: 501–510.
10. Kennerdell JR, Carthew RW. 1998. Use of dsRNA-mediated genetic interference to demonstrate that frizzled and frizzled 2 act in the wingless pathway. *Cell* **95**: 1017–1026.
11. Li YX, Farrell MJ, Liu R, Mohanty N, Kirby ML. 2000. Double-stranded RNA injection produces null phenotypes in zebrafish. *Dev Biol* **217**: 394–405.
12. Nasevicius A, Ekker SC. 2000. Effective targeted gene knock-down in zebrafish. *Nature Genet* **26**: 216–220.
13. Oates AC, Bruce AE, Ho RK. 2000. Too much interference: injection of double-stranded RNA has nonspecific effects in the zebrafish embryo. *Dev Biol* **224**: 20–28.
14. Oates AC, Wollberg P, Pratt SJ, *et al*. 1999. Zebrafish stat3 is expressed in restricted tissues during embryogenesis and stat1 rescues cytokine signalling in a STAT1-deficient human cell line. *Dev Dyn* **215**: 352–370.
15. Postlethwait J, Amores A, Force A, Yan YL. 1999. The zebrafish genome. *Methods Cell Biol* **60**: 149–163.
16. Qin G, Taylor M, Ning YY, Iversen P, Kobzik L. 2000. *In vivo* evaluation of a morpholino antisense oligomer directed against tumor necrosis factor-alpha. *Antisense Nucleic Acid Drug Dev* **10**: 11–16.
17. Raz E, van Luenen HG, Schaerringer B, Plasterk RHA, Driever W. 1998. Transposition of the nematode *Caenorhabditis elegans* Tc3 element in the zebrafish *Danio rerio*. *Curr Biol* **8**: 82–88.
18. Summerton J. 1999. Morpholino antisense oligomers: the case for an RNase H-independent structural type. *Biochim Biophys Acta* **1489**: 141–158.

Copyright © 2000 John Wiley & Sons, Ltd. 

19. Summerton J, Weller D. 1997. Morpholino antisense oligomers: design, preparation, and properties. *Antisense Nucleic Acid Drug Dev* **7**: 187–195.
20. Svoboda P, Stein P, Hayashi H, Schultz RM. 2000. Selective reduction of dormant maternal mRNAs in mouse oocytes by RNA interference. *Development* **127**: 4147–4156.
21. Talbot WS, Hopkins N. 2000. Zebrafish mutations and functional analysis of the vertebrate genome. *Genes Dev* **14**: 755–762.
22. Tavernarakis N, Wang SL, Dorovkov M, Ryazanov A, Driscoll M. 2000. Heritable and inducible genetic interference by double-stranded RNA encoded by transgenes. *Nature Genet* **24**: 180–183.
23. Wallis DE, Muenke M. 1999. Molecular mechanisms of holoprosencephaly. *Mol Genet Metabol* **68**: 126–138.
24. Wargelius A, Ellingsen S, Fjose A. 1999. Double-stranded RNA induces specific developmental defects in zebrafish embryos. *Biochem Biophys Res Commun* **263**: 156–161.
25. Wianny F, Zernicka-Goetz M. 2000. Specific interference with gene function by double-stranded RNA in early mouse development. *Nature Cell Biol* **2**: 70–75.
26. Zernicka-Goetz M. 2000. Jumping the gun on mouse gene expression. *Nature* **405**: 733.

*The* **Genomics** *Website at Wiley*

**www.wiley.co.uk/genomics**

The **Genomics** website at Wiley is a new and **DYNAMIC** resource for the genomics community, offering **FREE** special feature articles and new information **EACH MONTH**.

Find out more about *Comparative and Functional Genomics*, and how to view all articles published this year **FREE OF CHARGE**!

Visit the **Library** for hot books in Genomics, Bioinformatics, Molecular Genetics and more.

Click on **Primary Research** for information on all our up-to-the minute journals, including: *Genesis*, *Bioessays, Gene Function and Disease*, and the *Journal of Gene Medicine.*

Let the **Genomics** website at Wiley be your **guide** to genomics-related web sites, manufacturers and suppliers, and a calendar of conferences.

WILEY

1516

Copyright © 2000 John Wiley & Sons, Ltd. 

## References

[PDF_00277] Ekker S. C. (2000). Morphants: a new systematic vertebrate functional genomics approach.. Yeast.

[PDF_00425] Ekker S. C. (2000). Morphants: a new systematic vertebrate functional genomics approach.. Yeast.

[PDF_00407] Zernicka-Goetz M. (2000). Jumping the gun on mouse gene expression.. Nature.

